# Supplementary material for: Variation of connectivity across exemplar sensory and associative thalamocortical loops in the mouse
Source: eLife. 2020 Oct 26;9:e62554. doi: 10.7554/eLife.62554 (PMC7644223; doi:10.7554/eLife.62554)
Supplement: Transparent reporting form [file elife-62554-transrepform.pdf]

eLIFE
1st Floor
24 Hills Road
Cambridge CB2 1JP, UK
P 01223 855340
W elifesciences.org
T @elife

eLife's transparent reporting form

We encourage authors to provide detailed information *within their submission* to facilitate the interpretation and replication of experiments. Authors can upload supporting documentation to indicate the use of appropriate reporting guidelines for health-related research (see EQUATOR Network), life science research (see the BioSharing Information Resource), or the ARRIVE guidelines for reporting work involving animal research. Where applicable, authors should refer to any relevant reporting standards documents in this form.

If you have any questions, please consult our Journal Policies and/or contact us: editorial@elifesciences.org.

Sample-size estimation

- You should state whether an appropriate sample size was computed when the study was being designed
- You should state the statistical method of sample size computation and any required assumptions
- If no explicit power analysis was used, you should describe how you decided what sample (replicate) size (number) to use

Please outline where this information can be found within the submission (e.g., sections or figure legends), or explain why this information doesn't apply to your submission:

No explicit power analysis was used as the datasets generated in this study are for relative comparison of anatomical input-output connectivity. The sample size was chosen in accordance with standard practice for similar work.

Eg: Li P, Li SB, Wang X, Phillips CD, Schwarz LA, Luo L, de Lecea L, Krasnow MA. (2020) Brain Circuit of Claustrophobia-like Behavior in Mice Identified by Upstream Tracing of Sighing. *Cell Rep*. 31(11):107779.

Wall NR, Neumann PA, Beier KT, Mokhtari AK, Luo L, Malenka RC. (2019) Complementary Genetic Targeting and Monosynaptic Input Mapping Reveal Recruitment and Refinement of Distributed Corticostriatal Ensembles by Cocaine. *Neuron*.104(5):916-930.

Ren, J., Friedmann, D., Xiong, J., Liu, C. D., Ferguson, B. R., Weerakkody, T., DeLoach, K. E., Ran, C., Pun, A., Sun, Y., Weissbourd, B., Neve, R. L., Huguenard, J., Horowitz, M. A., & Luo, L. (2018). Anatomically Defined and Functionally Distinct Dorsal Raphe Serotonin Sub-systems. *Cell*, 175(2), 472–487.

Replicates

- You should report how often each experiment was performed
- You should include a definition of biological versus technical replication
- The data obtained should be provided and sufficient information should be provided to indicate the number of independent biological and/or technical replicates
- If you encountered any outliers, you should describe how these were handled
- Criteria for exclusion/inclusion of data should be clearly stated

eLife Sciences Publications, Ltd is a limited liability non-profit non-stock corporation incorporated in the State of Delaware, USA, with company number 5030732, and is registered in the UK with company number FC030576 and branch number BR015634 at the address 1st Floor, 24 Hills Road, Cambridge CB2 1JP | August 2014

- High-throughput sequence data should be uploaded before submission, with a private link for reviewers provided (these are available from both GEO and ArrayExpress)

Please outline where this information can be found within the submission (e.g., sections or figure legends), or explain why this information doesn't apply to your submission:

Throughout the manuscript animal numbers (biological) are reported as 'N' in figure legends while the technical replicates (individual neurons, sections) are referred to as 'n'. The biological replicates were always run in pairs of 1 each of $MD^{\rightarrow PL}$ and $MGB^{\rightarrow A1}$.

Eg: In Figure 3. The data was generated from 4 mice each for $MD^{\rightarrow PL}$ and $MGB^{\rightarrow A1}$. For each mouse 2 to 3 non-sequential sections were chosen to perform the analysis and the data from each section counted towards an individual datapoint (technical replicate). So, this experiment was repeated 4 times with 1 animal per condition.

This logic is consistent throughout the figure legends of the manuscript.

Statistical reporting

- Statistical analysis methods should be described and justified
- Raw data should be presented in figures whenever informative to do so (typically when N per group is less than 10)
- For each experiment, you should identify the statistical tests used, exact values of N, definitions of center, methods of multiple test correction, and dispersion and precision measures (e.g., mean, median, SD, SEM, confidence intervals; and, for the major substantive results, a measure of effect size (e.g., Pearson's r, Cohen's d)
- Report exact p-values wherever possible alongside the summary statistics and 95% confidence intervals. These should be reported for all key questions and not only when the p-value is less than 0.05.

Please outline where this information can be found within the submission (e.g., sections or figure legends), or explain why this information doesn't apply to your submission:

All datasets were assumed to not have normal distributions and hence non-parametric tests were used for significance testing throughout the manuscript. The statistical test used, and the p values are detailed in the figure legends of each dataset as well as in the Methods section under 'Statistical analysis'.

For data sets where the number of individual samples per condition was below 10, the raw data is plotted as dots and overlayed onto the summary plots as also mentioned in figure legends.

(For large datasets, or papers with a very large number of statistical tests, you may upload a single table file with tests, Ns, etc., with reference to sections in the manuscript.)

Group allocation

- Indicate how samples were allocated into experimental groups (in the case of clinical studies, please specify allocation to treatment method); if randomization was used, please also state if restricted randomization was applied

- Indicate if masking was used during group allocation, data collection and/or data analysis

Please outline where this information can be found within the submission (e.g., sections or figure legends), or explain why this information doesn't apply to your submission:

Animals were randomly assigned to undergo labelling of $MD^{\rightarrow PL}$ or $MGB^{\rightarrow A1}$ circuits for anatomy or physiology experiments and no distinction was made with respect to gender of the animals. This statement is outlined in the Method's section of the paper under 'Animals'

Additional data files ("source data")

- We encourage you to upload relevant additional data files, such as numerical data that are represented as a graph in a figure, or as a summary table
- Where provided, these should be in the most useful format, and they can be uploaded as "Source data" files linked to a main figure or table
- Include model definition files including the full list of parameters used
- Include code used for data analysis (e.g., R, MatLab)
- Avoid stating that data files are "available upon request"

Please indicate the figures or tables for which source data files have been provided:

eLife Sciences Publications, Ltd is a limited liability non-profit non-stock corporation incorporated in the State of Delaware, USA, with company number 5030732, and is registered in the UK with company number FC030576 and branch number BR015634 at the address 1st Floor, 24 Hills Road, Cambridge CB2 1JP | August 2014
